# Homocysteine Levels and Treatment Effect in the Prospective Study of Pravastatin in the Elderly at Risk

**DOI:** 10.1111/jgs.12660

**Published:** 2014-01-21

**Authors:** Yvonne M Drewes, Rosalinde K E Poortvliet, Jeanet W Blom, Wouter de Ruijter, Rudi G J Westendorp, David J Stott, Henk J Blom, Ian Ford, Naveed Sattar, J Wouter Jukema, Willem J J Assendelft, Anton J M de Craen, Jacobijn Gussekloo

**Affiliations:** a Department of Public Health and Primary Care, Leiden University Medical CenterLeiden, the Netherlands; b Department ofGerontology and Geriatrics, Leiden University Medical CenterLeiden, the Netherlands; c Faculty of Medicine, Academic Section of Geriatric Medicine, University of GlasgowGlasgow, UK; d Metabolic Unit, Department of Clinical Chemistry, Institute for Cardiovascular Research, VU University Medical CenterAmsterdam, the Netherlands; e Robertson Centre for Biostatistics, University of GlasgowGlasgow, UK; f Department of Vascular Biochemistry, University of GlasgowGlasgow, UK; g Department of Cardiology, Leiden University Medical CenterLeiden, the Netherlands; h Department of Primary Community Care, Radboud University Nijmegen Medical CentreNijmegen, the Netherlands

**Keywords:** homocysteine, older persons, cardiovascular risk, statins, prevention

## Abstract

**Objectives:**

To assess the effect of preventive pravastatin treatment on coronary heart disease (CHD) morbidity and mortality in older persons at risk for cardiovascular disease (CVD), stratified according to plasma levels of homocysteine.

**Design:**

A post hoc subanalysis in the PROspective Study of Pravastatin in the Elderly at Risk (PROSPER), started in 1997, which is a double-blind, randomized, placebo-controlled trial with a mean follow-up of 3.2 years.

**Setting:**

Primary care setting in two of the three PROSPER study sites (Netherlands and Scotland).

**Participants:**

Individuals (n = 3,522, aged 70–82, 1,765 male) with a history of or risk factors for CVD were ranked in three groups depending on baseline homocysteine level, sex, and study site.

**Intervention:**

Pravastatin (40 mg) versus placebo.

**Measurements:**

Fatal and nonfatal CHD and mortality.

**Results:**

In the placebo group, participants with a high homocysteine level (n = 588) had a 1.8 higher risk (95% confidence interval (CI) = 1.2–2.5, *P* = .001) of fatal and nonfatal CHD than those with a low homocysteine level (n = 597). The absolute risk reduction in fatal and nonfatal CHD with pravastatin treatment was 1.6% (95% CI = −1.6 to 4.7%) in the low homocysteine group and 6.7% (95% CI = 2.7–10.7%) in the high homocysteine group (difference 5.2%, 95% CI = 0.11–10.3, *P* = .046). Therefore, the number needed to treat (NNT) with pravastatin for 3.2 years for benefit related to fatal and nonfatal CHD events was 14.8 (95% CI = 9.3–36.6) for high homocysteine and 64.5 (95% CI = 21.4–∞) for low homocysteine.

**Conclusion:**

In older persons at risk of CVD, those with high homocysteine are at highest risk for fatal and nonfatal CHD. With pravastatin treatment, this group has the highest absolute risk reduction and the lowest NNT to prevent fatal and nonfatal CHD.

The aim of cardiovascular risk management is to reduce the incidence of cardiovascular events in high-risk populations. Clinical cardiovascular risk scores, such as the Framingham Risk Score^1^ and the Systematic Coronary Risk Evaluation (SCORE),^2^ are used worldwide to select those with high cardiovascular risk, but their accuracy to predict risk of cardiovascular outcomes declines with advancing age.^3^–^6^ Because the prevalence and incidence of cardiovascular disease (CVD) increases exponentially with age,^7^–^9^ some have suggested that preventive treatment be offered to everyone over a specified age without measuring other risk factors,^10^ but others emphasize the need for risk stratification in old age.^11^,^12^ Recently, the Leiden 85-plus Study (and others) showed that homocysteine is predictive of cardiovascular events in old age.^13^–^18^

Because risk predictors are clinically meaningful only when effective preventive treatment is available,^19^ which treatment possibilities exist (and are appropriate) for older persons with high homocysteine to lower their cardiovascular risk needs to be established. Large randomized controlled trials (RCTs) and meta-analyses show that lowering plasma homocysteine using treatment with folate has no beneficial effect on the incidence of cardiovascular events.^16^,^20^,^21^ The PROspective Study of Pravastatin in the Elderly at Risk (PROSPER)^22^ has shown that pravastatin lowers the risk of coronary heart disease (CHD) in older people in general, but not the risk of fatal or nonfatal stroke. The current study was designed to determine whether older persons with high homocysteine levels would benefit more from this conventional preventive treatment than those with lower levels.

A post hoc subanalysis was performed in PROSPER (a large double-blind, randomized, placebo-controlled trial) to assess the effect of pravastatin on CHD risk and mortality in older persons, stratified according to plasma level of homocysteine.

## Methods

### Study Design

The protocol of PROSPER has been published elsewhere.^23^ Briefly, in 1997 to 1999, 5,804 individuals were enrolled in Scotland (n = 2,520), Ireland (n = 2,184), and the Netherlands (n = 1,100). Men and women aged 70 to 82 were recruited, with preexisting vascular disease (coronary, cerebral, or peripheral) or at risk of such disease because of smoking, hypertension, or diabetes mellitus. Their plasma total cholesterol was required to be 154 to 347 mg/dL (4.0–9.0 mM) and their triglyceride concentrations 531 mg/dL or less (≤6.0 mM). Individuals with congestive heart failure (New York Heart Association functional class III or IV) or poor cognitive function (Mini-Mental State Examination (MMSE) score <24 points) were excluded. Participants were randomized to a group receiving 40 mg of pravastatin a day or to a control group receiving placebo and were followed (on average) for 3.2 years. Throughout the study, all study personnel were unaware of the allocated study medication status of the participants. The institutional ethics review boards of all centers approved the protocol, and all participants gave written informed consent.

### Determination of Homocysteine

After blood was drawn, blood samples were kept at room temperature until they were processed in the laboratory to be stored in the biobank (−80°C). In 2010, homocysteine concentrations were measured in the biobank ethylenediaminetetraacetic acid plasma samples, from samples taken at baseline (blood samples, n = 5,757; missing, n = 47) and again at 6 months. Measurements were done in batches after reduction to the free form with a fluorescence polarization immunoassay (IMx analyzer; Abbott, Abbott Park, IL).

The median plasma homocysteine level was 14.1 μM (interquartile range (IQR) 11.8–17.0) in the Netherlands (n = 1,100), 17.9 μM (IQR 15.3–21.8) in Scotland (n = 2,505), and 18.8 μM (IQR 15.6–23.3) in Ireland (n = 2,152), although there were differences in standard operating procedures between the study sites. (The Dutch and Scottish blood samples were processed within 8 hours, whereas in Ireland, this processing frequently exceeded 8 hours.) Statistical analysis showed that the variance in log homocysteine for Scotland and the Netherlands was comparable (*F* = 2.4, *P* = .12), but both were significantly different from Ireland (Scotland vs Ireland, *F* = 5.4, *P* = .02; the Netherlands vs Ireland, *F* = 11.2, *P* = .001). Because plasma homocysteine levels increase by 0.5 to 1.0 μM/h in blood at room temperature,^24^–^26^ differences in lag time could explain the differences in variance between Ireland and the other countries. Therefore, it was decided to exclude participants from Ireland from this analysis.

### Outcomes

The outcomes, described in the design of PROSPER,^23^ were the incidence of fatal and nonfatal CHD (including definite or suspected CHD mortality and nonfatal myocardial infarction (MI)), nonfatal MI, CHD mortality, non-CHD mortality, and all-cause mortality. The PROSPER Endpoints Committee, which was blinded to study medication and plasma levels of homocysteine, assessed all CHD endpoints.

### Data Analysis

At baseline, participants were ranked in three groups equal in size (low, medium, and high homocysteine) based on plasma homocysteine level, sex, and study site. Within each homocysteine group, the baseline characteristics of the placebo and treatment groups were compared using independent *t*-tests for continuous data and Pearson chi-square tests (degrees of freedom (df) = 1) for categorical data.

#### Predictive Value of Homocysteine in the Placebo Group

The cumulative incidence rates of fatal and nonfatal CHD and all-cause mortality for the three homocysteine groups were calculated using Kaplan–Meier curves and compared using the log rank test (df = 2). Hazard ratios (HRs) and corresponding 95% confidence intervals (95% CIs) for sex- and study site–specific tertiles of homocysteine were calculated for the endpoints using Cox proportional hazard models (reference low homocysteine), with adjustment for age. To further investigate the independent predictive value of homocysteine, baseline history of CVD, baseline Framingham risk factors (smoking, diabetes mellitus, left ventricle hypertrophy, systolic blood pressure, total cholesterol, high density lipoprotein cholesterol (HDL-C)), and earlier published predictors in PROSPER (C-reactive protein (CRP), and creatinine clearance (Cockcroft-Gault)) were also adjusted for.

#### Treatment Effect Comparing the Placebo and Treatment Groups

The treatment effects of pravastatin and placebo according to homocysteine group were calculated using two methods. First, according to homocysteine group, the cumulative incidence rate for fatal and nonfatal CHD and all-cause mortality are presented for those taking placebo and those taking pravastatin with Kaplan–Meier curves and compared using the log rank test (df = 1) and Cox proportional hazard models. No adjustments were made. The presence of multiplicative interaction was formally tested by adding the interaction term (treatment × homocysteine group) to the Cox regression model. All analyses were on an intention-to-treat basis.

Second, the absolute risk reduction according to treatment with pravastatin was calculated. Differences in absolute risk reductions between the homocysteine groups were tested using a *z*-test. Numbers needed to treat (NNT) to benefit were calculated over the mean follow-up of the trial (3.2 years) based on the difference in cumulative proportion surviving in the placebo and pravastatin groups.^27^,^28^ Because creatinine clearance seems to be associated with homocysteine levels, a subgroup analysis for absolute risk reduction was conducted for fatal and nonfatal CHD and for all-cause mortality in participants with creatinine clearance of 30 mL/min or greater.

#### Influence of Treatment with Pravastatin on Homocysteine

To investigate whether pravastatin treatment influences plasma homocysteine levels, homocysteine concentrations were measured after 6 months of treatment for 1,832 participants (183 taking placebo, 1,649 taking pravastatin). The effect of pravastatin treatment on plasma homocysteine levels was tested after 6 months using linear regression analysis adjusted for baseline homocysteine.

## Results

In total, 3,620 PROSPER participants from the Netherlands and Scotland were eligible for this study. Because 15 participants had missing biobank samples, and 83 had missing homocysteine measurements, 3,522 participants (1,764 placebo, 1,758 pravastatin) were included in the analyses. The cutoff values of the homocysteine tertiles (33% and 67%) in the Netherlands (n = 1,049) were 11.7 and 14.7 μM, respectively, for women and 13.5 and 16.9 μM, respectively, for men; for Scotland (n = 2,473) these limits were 15.4 and 19.6 μM, respectively, for women, and 16.9 and 21.0 μM, respectively, for men.

### Baseline Characteristics

Table 1 presents baseline characteristics of the total group of participants and according to homocysteine group, stratified for placebo or pravastatin. In the total group, the mean age of participants was 75.3 ± 3.4, and 48% had a history of CVD. Mean homocysteine level at baseline was 18.3 ± 7.1 μM. There were no differences according to homocysteine group in baseline characteristics between the pravastatin and placebo groups. The proportion of participants with diabetes mellitus was lower in the high homocysteine group.

**Table 1:** Baseline Participant Characteristics According to Treatment and Homocysteine Level (n = 3,522)

		Homocysteine Level					
		Low		Medium		High	
Characteristic	All	Placebo, n = 597	Pravastatin, n = 575	Placebo, n = 579	Pravastatin, n = 598	Placebo, n = 588	Pravastatin, n = 585
Demographic and functional characteristics							
Study site Scotland, n (%)	2,473 (70)	424 (71)	400 (70)	400 (69)	425 (71)	416 (71)	408 (70)
Male, n (%)	1,765 (50)	296 (50)	291 (51)	286 (49)	304 (51)	285 (49)	303 (52)
Age, mean ± SD	75.3 ± 3.4	74.9 ± 3.4	74.9 ± 3.3	75.2 ± 3.2	75.2 ± 3.3	75.6 ± 3.5	75.7 ± 3.4
Mini-Mental State Examination score, mean ± SD	28.2 ± 1.5	28.3 ± 1.4	28.3 ± 1.4	28.3 ± 1.4	28.2 ± 1.5	28.1 ± 1.5	28.0 ± 1.6
Barthel index, mean± SD	19.8 ± 0.7	19.7 ± 0.7	19.8 ± 0.8	19.8 ± 0.7	19.8 ± 0.5	19.7 ± 0.8	19.7 ± 0.9
Instrumental activity of daily living score, mean ± SD	13.6 ± 0.9	13.6 ± 0.9	13.6 ± 0.9	13.7 ± 0.9	13.7 ± 0.7	13.6 ± 1.1	13.5 ± 1.0
Clinical history and cardiovascular risk factors							
History of cardiovascular disease^a^	1,675 (48)	267 (45)	272 (47)	269 (47)	279 (47)	298 (51)	290 (50)
History of diabetes mellitus	386 (11)	88 (15)	85 (15)	68 (12)	57 (9.5)	45 (7.7)	43 (7.4)
Creatinine clearance <30 mL/min^b^	96 (2.7)	12 (2.0)	15 (2.6)	14 (2.4)	16 (2.7)	17 (2.9)	22 (3.8)
Body mass index, kg/m^2^, mean ± SD	27.0 ± 5.5	26.9 ± 5.6	26.9 ± 5.5	27.3 ± 5.7	27.0 ± 5.3	27.2 ± 5.6	26.8 ± 5.4
Current smoker	943 (27)	157 (26)	137 (24)	156 (27)	166 (28)	167 (28)	160 (27)
Alcohol, U/wk, mean ± SD^c^	5.3 ± 8.4	4.9 ± 7.3	5.0 ± 7.5	5.5 ± 8.7	5.6 ± 9.5	4.9 ± 7.7	5.7 ± 9.4
Systolic blood pressure, mmHg, mean ± SD	154.7 ± 21.4	153.4 ± 20.6	154.1 ± 21.1	156.0 ± 20.5	154.2 ± 21.2	155.4 ± 23.1	155.2 ± 21.6
Total cholesterol, mg/dL, mean ± SD	220.7 ± 35.5	218.4 ± 34.9	219.6 ± 35.8	220.8 ± 33.9	221.5 ± 35.2	220.2 ± 36.0	223.6 ± 36.9
Low-density lipoprotein cholesterol, mg/dL, mean ± SD	148.5 ± 31.0	146.8 ± 30.9	147.6 ± 30.3	148.9 ± 29.8	148.6 ± 31.1	147.9 ± 32.1	151.1 ± 32.0
High-density lipoprotein cholesterol, mg/dL, mean ± SD	49.2 ± 13.4	49.8 ± 12.6	48.8 ± 13.0	49.2 ± 13.0	49.3 ± 13.1	49.2 ± 14.5	49.3 ± 14.1
Triglycerides, mg/dL, mean ± SD	136.5 ± 61.2	131.4 ± 59.1	138.2 ± 63.4	136.1 ± 58.5	140.4 ± 66.2	136.5 ± 60.8	136.5 ± 58.4
Homocysteine, μM, mean ± SD	18.3 ± 7.1	13.0 ± 2.1	13.1 ± 2.1	16.8 ± 2.2	17.0 ± 2.2	25.2 ± 8.6	24.6 ± 8.2

SD = Standard Deviation.

a Stable angina pectoris, intermittent claudication, stroke, transient ischemic attack, myocardial infarction, peripheral arterial disease surgery, or amputation for vascular disease ≥6 months before study entry.

b Calculated using the Cockroft-Gault formula.

c 1 U = 60 mL distilled spirits, 170 mL wine, or 300 mL beer.

### Predictive Value of Homocysteine in the Placebo Group

**Figure 1:**
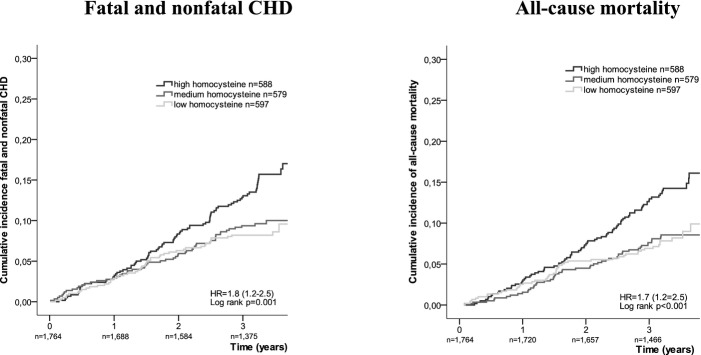
Cumulative incidence of fatal and nonfatal coronary heart disease (CHD) and all-cause mortality depending on baseline plasma levels of homocysteine in the placebo group (n = 1,764). HR = Hazard Ratio; CI = Confidence Interval: high versus low homocysteine group, adjusted for age.

Figure 1 shows the cumulative incidence of fatal and nonfatal CHD and all-cause mortality for the three homocysteine groups in the placebo group. Participants with medium homocysteine levels had no greater risk of fatal and nonfatal CHD than those with low homocysteine (HR = 1.1, 95% CI = 0.76–1.6, *P* = .57), but those with high homocysteine had a 1.8 times greater risk (95% CI = 1.2–2.5, *P* = .001). For overall mortality, the HRs were 1.0 (95% CI = 0.67–1.5, *P* = .99) and 1.7 (95% CI = 1.2–2.5, *P* = .003), respectively. These estimates did not change after additional adjustments for history of CVD; Framingham risk factors; or CRP, HDL-C, and creatinine clearance (data not shown).

Similarly, participants with high homocysteine levels had a greater risk of nonfatal MI, CHD mortality, and non-CHD mortality. Furthermore, no differences in risk were found between the medium and low homocysteine level groups for any of these outcomes (data not shown).

#### Treatment Effect Depending on Homocysteine

**Figure 2:**
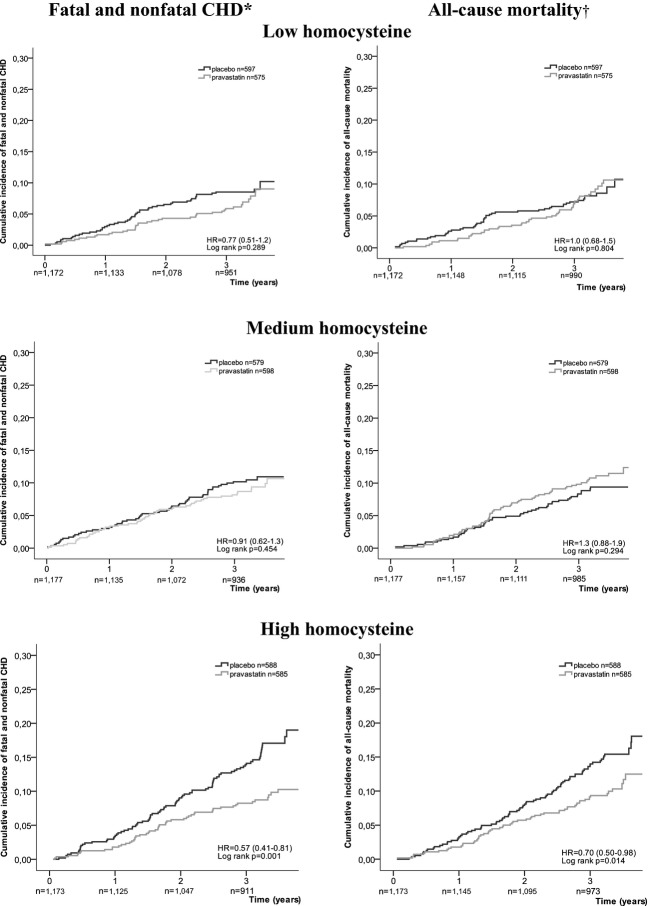
Cumulative incidence fatal and nonfatal coronary heart disease (CHD) and all-cause mortality depending on pravastatin treatment, stratified according to plasma homocysteine level at baseline. *P for multiplicative interaction = .21, †*P* for multiplicative interaction = .10.

Figure 2 presents the cumulative incidence of fatal and nonfatal CHD and all-cause mortality in participants with and without pravastatin according to homocysteine group. In participants with high homocysteine, an HR of 0.57 (95% CI = 0.41–0.81, *P* = .002) was found as the treatment effect of pravastatin on fatal and nonfatal CHD, and an HR of 0.70 (95% CI = 0.50–0.98, *P* = .04) as the treatment effect on all-cause mortality. With medium and low homocysteine levels, there was no significant difference in cumulative incidence between placebo and pravastatin treatment.

Moreover, multiplicative interaction was not found between treatment and homocysteine group (fatal and nonfatal CHD: *P* for multiplicative interaction = .208, all-cause mortality: *P* for multiplicative interaction = .097; Figure 2). Similar patterns were seen for nonfatal MI and CHD mortality. For non-CHD mortality, no effect of treatment with pravastatin was found in any of the three homocysteine groups (data not shown).

Table 2 presents absolute treatment effects of pravastatin according to homocysteine group. The absolute risk reduction in fatal and nonfatal CHD with pravastatin treatment was 1.6% (95% CI = −1.6 to 4.7) in the low homocysteine group and 6.7% (95% CI = 2.7–10.7) in the high homocysteine group (absolute risk reduction difference = 5.2%, 95% CI = 0.11–10.3, *P* = .046). For all-cause mortality, the absolute risk reductions were −0.66% (95% CI = −4.0 to 2.7) and 4.6% (95% CI = 0.78–8.4), respectively (absolute risk reduction difference = 5.2%, 95% CI = 0.19–10.3, *P* = .04).

**Table 2:** Absolute Effect of Treatment with Pravastatin on Cardiovascular Outcomes and Mortality After 3.2 Years According to Homocysteine Level

	Placebo		Pravastatin		ARR (95% CI)	Difference in ARR (vs Low) (95% CI)	*P*-Value^b^
Outcomes and Homocysteine Groups^a^	n	Cumulative Incidence of Events,% (95% CI)	n	Cumulative Incidence of Events,% (95% CI)			
Fatal and nonfatal CHD							
Low	48	8.2 (6.0–10.5)	32	6.7 (4.5–8.9)	1.6 (−1.6–4.7)	Reference	
Medium	54	9.9 (7.4–12.4)	47	8.7 (6.3–11.0)	1.2 (−2.2–4.7)	−0.32 (−5.0–4.3)	.89
High	76	15.9 (12.8–19.1)	47	9.2 (6.7–11.7)	6.7 (2.7–10.7)	5.2 (0.11–10.3)	.046
Nonfatal MI							
Low	29	5.1 (3.3–6.9)	26	5.6 (3.6–7.7)	−0.5 (−3.2–2.2)	Reference	
Medium	39	7.3 (5.1–9.5)	26	5.1 (3.2–7.0)	2.2 (−0.73–5.0)	2.7 (−1.3–6.7)	.18
High	51	11.3 (8.5–14.1)	32	6.4 (4.3–8.5)	4.9 (1.4–8.4)	5.5 (1.0–9.9)	.02
CHD mortality							
Low	20	3.4 (1.9–4.9)	11	2.0 (0.83–3.2)	1.4 (–0.45–3.3)	Reference	
Medium	18	3.3 (1.8–4.7)	27	4.7 (3.0–6.4)	−1.5 (−3.7–0.83)	−2.9 (−5.8–0.08)	.06
High	33	6.5 (4.4–8.6)	18	3.5 (1.9–5.1)	3.0 (0.35–5.6)	1.5 (−1.7–4.8)	.35
Non-CHD mortality							
Low	25	4.8 (3.0–6.6)	31	6.9 (4.6–9.1)	−2.1 (−4.9–0.84)	Reference	
Medium	30	5.4 (3.5–7.3)	32	5.9 (3.9–7.8)	−0.46 (−3.2–2.3)	1.6 (−2.4–5.6)	.43
High	46	8.4 (6.1–10.7)	34	6.5 (4.4–8.5)	2.0 (−1.2–5.1)	4.0 (−0.24–8.2)	.06
All-cause mortality							
Low	45	8.0 (5.8–10.3)	42	8.7 (6.3–11.1)	−0.66 (−4.0–2.7)	Reference	
Medium	48	8.5 (6.2–10.8)	59	10.3 (7.8–12.8)	−1.8 (−5.2–1.6)	−1.1 (−5.9–3.6)	.64
High	79	14.3 (11.4–17.2)	52	9.8 (7.3–12.2)	4.6 (0.78–8.4)	5.2 (0.19–10.3)	.04

CI = confidence interval; CHD = coronary heart disease.

a Group sizes: low: placebo n = 597, pravastatin n = 575; medium: placebo n = 579, pravastatin n = 598; high: placebo n = 588, pravastatin n = 585.

b *P*-value of difference in absolute risk reduction (ARR) compared with reference group low homocysteine estimated using *z*-test.

Mean homocysteine values of 18.3 μM were found in persons with creatinine clearance of 30 mL/min or greater (n = 3,426) and of 19.0 μM (*P* = .29) in persons with creatinine clearance of <30 mL/min (n = 96). Because creatinine clearance is known to be associated with homocysteine level, a subgroup analysis was conducted in persons with creatinine clearance of 30 mL/min or greater. The differences in absolute risk reductions according to pravastatin treatment remained similar (fatal and nonfatal CHD 6.0%, 95% CI = 0.84–11.1, *P* = .02; all-cause mortality 5.8%, 95% CI = 0.70–11.0, *P* = .03).

**Figure 3:**
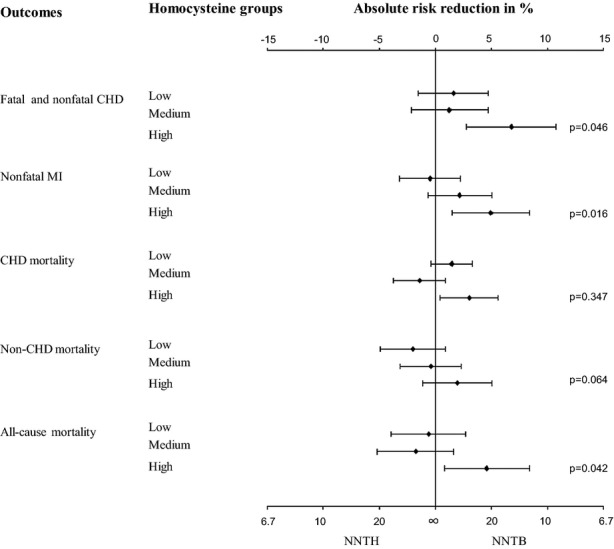
Number needed to treat (NNT) with pravastatin after 3.2 years according to homocysteine level at baseline. *P*-value of difference between high and low homocysteine group for absolute risk reduction in% and for NNT estimated using *z*-test. NNTH = NNT to Harm; NNTB = NNT to Benefit; CHD = Coronary Heart Disease; MI = Myocardial Infarction.

For fatal and nonfatal CHD, the NNT with pravastatin to benefit for 3.2 years was 14.8 (95% CI = 9.3–36.6) in the high homocysteine group, 81.3 (95% CI = 21.5–∞) in the medium homocysteine group, and 64.5 (95% CI = 21.4–∞) in the low homocysteine group (high vs low, *P* = .046) (Figure 3). For all-cause mortality, a beneficial result was found in the high homocysteine group (NNT = 21.8, 95% CI = 11.9–129) but no benefit in the medium and low homocysteine groups (high vs low, *P* = .04).

#### Influence of Pravastatin on Homocysteine

After 6 months of treatment, homocysteine levels had fallen −0.52 μM (95% CI = −1.1 to 0.07) from baseline for those on pravastatin (linear regression, *P* = .08).

## Discussion

This study suggests that homocysteine may be a promising new CHD risk predictor in older people, because high plasma homocysteine levels not only indicate older persons at high risk for fatal and nonfatal CHD and all-cause mortality, but also identify those with the highest absolute risk reduction due to the use of pravastatin and the lowest NNT to prevent fatal and nonfatal CHD.

Earlier studies showed that older persons with high homocysteine levels are at greater risk of cardiovascular events, and homocysteine level may provide additional risk stratification beyond traditional risk factors.^13^–^18^ For example, a previous study examined whether adding homocysteine to a model based on traditional CVD risk factors improved classification. In two younger population cohorts, they found that addition of homocysteine level to Framingham risk score significantly improved risk prediction.^18^ Moreover, for persons aged 85 and older, a previous study showed that the classic risk factors as included in the Framingham risk score no longer accurately predicted cardiovascular mortality in those with no history of CVD, whereas a single measurement of homocysteine accurately identified those at high risk of cardiovascular mortality.^13^ The findings of the current study not only confirm studies reporting that homocysteine predicts CHD risk in old age, but also show the independent predictive capacity in a selected population of older persons with high cardiovascular risk. A recent metaanalysis showed that a moderate homocysteine elevation due to genetic variance does not meaningfully affect CHD risk.^29^ This finding indicates that circulating homocysteine levels within the normal range are not causally related to CHD risk. Moreover, large RCTs and their meta-analyses show that lowering plasma homocysteine by treatment with folate has no beneficial effect on the incidence of cardiovascular events.^16^,^20^,^21^ Therefore, the underlying biological pathway to explain the predictive value of high homocysteine levels for CVD, if there is one, is unknown. Homocysteine may be seen as an epiphenomenon rather than a causal agent, but this does not refute its predictive abilities.

Because homocysteine was found to be not causally related to CVD, whether preventive treatment could be offered to those with high homocysteine to reduce their cardiovascular risk is unknown. In the Air Force/Texas Coronary Atherosclerosis Prevention Study (AFCAPS/TexCAPS),^30^ with only a small proportion of older adults, the beneficial effect of statin treatment in people with high homocysteine levels was limited to people with a low-density lipoprotein cholesterol (LDL-C) level higher than 149 mg/dL. There results further extend the findings from the AFCAPS/TexCAPS by demonstrating that the benefits of statin treatment may differ according to homocysteine level in high-risk individuals with an average LDL-C level of 148 mg/dL. If these findings hold true in a subsequent study, there could be a role for measurement of homocysteine levels to help guide decisions on statin use in older adults, which is a widely available treatment. This is an important criterion underlying screening.^19^

The present study revealed other findings that deserve further examination. First, although a clear CHD risk benefit from pravastatin therapy over the trial period of 3.2 years was found only in older persons with high and not those with low and medium homocysteine levels, a multiplicative interaction for the treatment effect was not found. Therefore, there is a possibility that pravastatin has the same treatment effect in all three homocysteine groups, although even when the relative treatment effect is similar between these groups, those at highest absolute risk will have the most benefit in terms of absolute risk reduction. This absolute risk reduction and corresponding NNT is important in clinical practice and guidelines, because it indicates the number of persons who need to be treated to prevent one event.

Second, the effect of pravastatin in the homocysteine groups does not show a linear trend. A lack of power because of a small number of events in the low and medium homocysteine level groups could explain this finding, so the possibility of random variation cannot be excluded, although it is also possible that pravastatin therapy is effective only in people with homocysteine levels greater than a certain cutoff value. The possibility of absolute cutoff values requires more in-depth study, investigated in a population with consistent blood sampling and storage procedures.

Third, it was found that plasma levels of homocysteine did not change significantly with pravastatin treatment over a 6-month period, although a small reduction was seen. Further examination is needed to determine the effect of pravastatin treatment on homocysteine levels. If pravastatin does not affect homocysteine levels, homocysteine measurement might be useful to evaluate the need for continuing preventive cardiovascular therapy in persons under pravastatin treatment.

For new biomarkers, others have investigated whether high cardiovascular risk and corresponding benefit from treatment can be predicted. A large-scale RCT^31^ and an earlier analysis in PROSPER^32^ showed that baseline CRP concentration predicts cardiovascular risk but not the relative CHD risk benefits of pravastatin therapy. Other analyses in PROSPER showed that HDL-C^33^ and creatinine clearance^34^ can predict benefit from pravastatin therapy for prevention of fatal and nonfatal CHD, although a high plasma homocysteine level remained predictive of a beneficial effect of pravastatin even in persons with creatinine clearance of 30 mL/min or greater. Furthermore, pravastatin was more effective in preventing cardiovascular events in those without diabetes mellitus.^22^ The current study showed that adjustment for these predictors did not influence the predictive value of homocysteine. A next step is to study the clinical value of homocysteine and other biomarkers by comparing their predictive value in combination with treatment response to develop the most-effective predictors of cardiovascular risk and treatment benefit. This is particularly important because statins are not without side effects or costs, and targeting those most at risk and with the most to gain would be clinically and economically advantageous.

### Strength and Limitations

This study was embedded in PROSPER, a large double-blind, randomized, placebo-controlled trial in older persons. This landmark clinical cardiovascular trial was performed following guidelines of good clinical practice, including endpoints that the Endpoint Committee uniformly assessed. Because homocysteine was assessed after closure of the trial, plasma levels of homocysteine had no influence on the study procedures, clinical treatment during follow-up, or the decisions of the Endpoint Committee.

A limitation of this study is that PROSPER procedures were not originally designed to collect optimal blood samples for the assessment of plasma homocysteine. Therefore, data could be used from only two of the three PROSPER study sites, although it is possible that some samples from those two sites were stored at room temperature for up to 8 hours before they were processed in the laboratory. This could have led to artificially high homocysteine plasma levels and therefore to misclassification in a higher homocysteine group. Because it was assumed that the samples with artificially high homocysteine levels are spread nondifferential in the population, this could have resulted in underestimation of the differences in treatment effect by homocysteine levels. Because it is also known that homocysteine levels vary between countries,^13^,^35^,^36^ more studies are needed to validate the absolute cutoff values of homocysteine to select elderly adults at highest risk in clinical practice.

Moreover, data regarding the use of B vitamins, which lower homocysteine levels, are not available. Another limitation of this study is that the treatment-by-homocysteine group multiplicative interaction was not significant, although the difference in absolute risk reduction between high and low homocysteine was significant (*P* = .046 for fatal and nonfatal CHD and *P* = .04 for all-cause mortality). The possibility of a type 1 error from multiple comparisons cannot be excluded.

It might also be seen as a limitation that these analyses focused only on homocysteine level to predict CHD risk and treatment effect, rather than investigating the etiological mechanisms behind the findings. Predictive and etiological studies will contribute to the further development of cardiovascular risk management in older adults, both on their own merits.

### Implications

A recent analysis of cost-effectiveness of statin treatment in primary care showed that, even in older adults, it is useful to stratify for risk of cardiovascular outcomes.^12^ The current study shows that homocysteine may predict CHD risk in the PROSPER population of older adults with high cardiovascular risks. As a consequence, individuals without traditional risk factors and thus with the lowest risks were excluded. Before these results can be implemented in current guidelines, further research is needed to find a cutoff value of homocysteine and confirm that high-risk older adults with high homocysteine levels obtain more benefit from pravastatin treatment. Moreover, whether homocysteine is useful in predicting benefits from pravastatin treatment for low- or intermediate-risk older adults remains to be investigated.

Homocysteine is a promising CHD risk predictor in old age, not only because high plasma homocysteine identifies older adults at high risk of fatal and nonfatal CHD, but also because those persons have the highest absolute risk reduction with pravastatin treatment and the lowest NNT to prevent fatal and nonfatal CHD. This is an important step in the further development of CHD risk stratification and treatment for older people.
